# World Heart Federation Policy Brief: Front-Of-Pack Labelling: Unhealthy Changes in the Global Food System

**DOI:** 10.5334/gh.935

**Published:** 2020-10-16

**Authors:** Beatriz Champagne, Monika Arora, Ahmed ElSayed, Susanne Løgstrup, Pamela Naidoo, Trevor Shilton, Diana Vaca McGhie, Kelcey Armstrong-Walenczak, Florence Berteletti, Sandya Ganesan, Barry Popkin

**Affiliations:** 1 Coalición Latinoamérica Saludable/Healthy Latin America Coalition, US; 2 Public Health Foundation of India, IN; 3 Alzaiem Alazhari University, SD; 4 European Heart Network; 5 Heart and Stroke Foundation South Africa, ZA; 6 National Heart Foundation of Australia, AU; 7 American Heart Association, US; 8 World Heart Federation, CH; 9 University of North Carolina, US

**Keywords:** nutrition, food policy, front-of-pack labeling, world food day, obesity

## Abstract

On World Food Day, the World Heart Federation calls on governments to implement mandatory front-of-pack food labels. The World Heart Federation (WHF) has developed a new policy brief on front-of-pack labelling (FOPL) aimed at improving global standards on nutrition and creating healthy food environments. Poor diet is responsible for more deaths worldwide than any other risk factor, and is a leading cause of obesity, type 2 diabetes, and cardiovascular disease (CVD). Global estimates suggest that almost 2.3 billion children and adults are overweight. The growing availability of ultra-processed foods, which contain high levels of sugars, sodium, saturated fats and refined carbohydrates, is a key contributor to the current obesity epidemic, which is increasingly impacting low- and middle-income countries. The WHF Front-of-Pack Labelling Policy Brief highlights front-of-pack labelling as a way to create environments where consumers are able to make better informed, healthier food choices for themselves and their families. Currently, a wide variety of front-of-pack labelling systems have been implemented by governments and food manufacturers around the world, with varying levels of success. The new WHF Policy Brief provides evidence-based, practical guidance that can be adapted to local contexts. It highlights that in order the be implemented successfully, FOPL systems must take into account consumer literacy and prevailing cultural norms around food and nutrition. FOPL must be mandatory, government-led, and accompanied by broad public nutrition education initiatives. The WHF Policy Brief includes a set of policy recommendations to give governments the tools they need to select the FOPL system that will best meet the needs of their populations, including recommendations on how to develop an effective FOPL programme, how to implement it successfully, and how to monitor and evaluate outcomes.

## Front-of-Pack Labelling (FOPL) and Cardiovascular Disease (CVD)

### CVD remains the leading cause of death worldwide

Together with hypertension, tobacco use, and physical inactivity, obesity is a known risk factor for CVD that can make changes to heart structure and function due to build-up of fat tissue. Because of these adverse effects, the World Heart Federation (WHF) works to combat obesity, as it is a barrier in achieving our vision of health justice and heart health for everyone. WHF is dedicated to promoting health behaviours and initiatives that will curb the global obesity epidemic.

Solutions like FOPL are necessary to improve the diets of consumers everywhere and to decrease risk factors for CVD.^1^,^2^

## WHY IS FOPL IMPORTANT?

### FOPL can enable consumers to make informed and healthier choices

FOPL systems should empower customers to make healthier choices and contribute to the prevention of the most concerning diet-related health conditions in a country. Depending on the priority, the main purpose of FOPL can be achieved by quickly informing consumers about the relative healthiness of products or by warning them when products are excessive in added sugars, total fat, saturated fats, trans fats and/or sodium, which are the critical nutrients associated with top risk factors for the most burdensome diseases in many countries (e.g. heart disease, high blood pressure, high fasting plasma glucose, and overweight and obesity).

### Consumers need help making healthy choices and identifying harmful products

The sheer number of products available makes shopping for food a confusing process for consumers. Having so many different types of foods, from both domestic and foreign manufacturers, makes it difficult for consumers to select nutritious products, even if an increasing number of them want to make healthy choices.^3^ Most shoppers spend fewer than ten seconds selecting each item, which does not give them enough time to compare the nutrition facts panels that are found on the backs of packaged foods.

Consumers are instead made to compare different types of FOPL from various manufacturers and regions that can be based on different values or use hard-tointerpret symbols.

Due to lack of understanding of the varied FOPL systems, consumers can be misled to purchase unhealthy options.^4^ Additionally, unhealthy products may feature misleading nutrition claims on their packages. Claims related to the amount of a specific nutrient in the food and direct or indirect claims about a food’s potential health benefits can give an unhealthy product a “health halo effect” and can cause consumers to misunderstand its nutritional quality.

**Figure:**
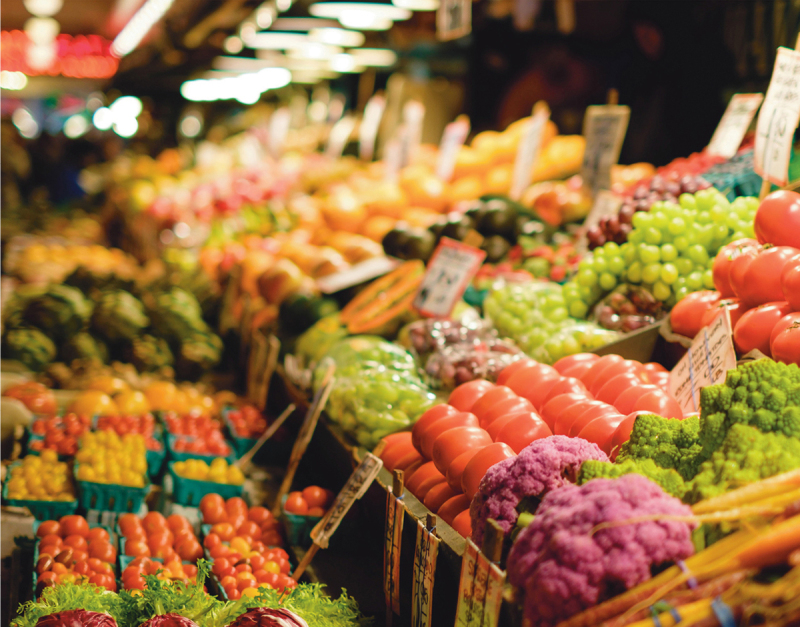


### Ultra-processed food products (UPP) have become increasingly available across the world

UPP are frequently high in calories and have little nutritional value. The great majority of these pre-packaged foods are ultra-processed with high levels of added sugars, sodium (salt), saturated fats, and refined carbohydrates.^5^

**Figure:**
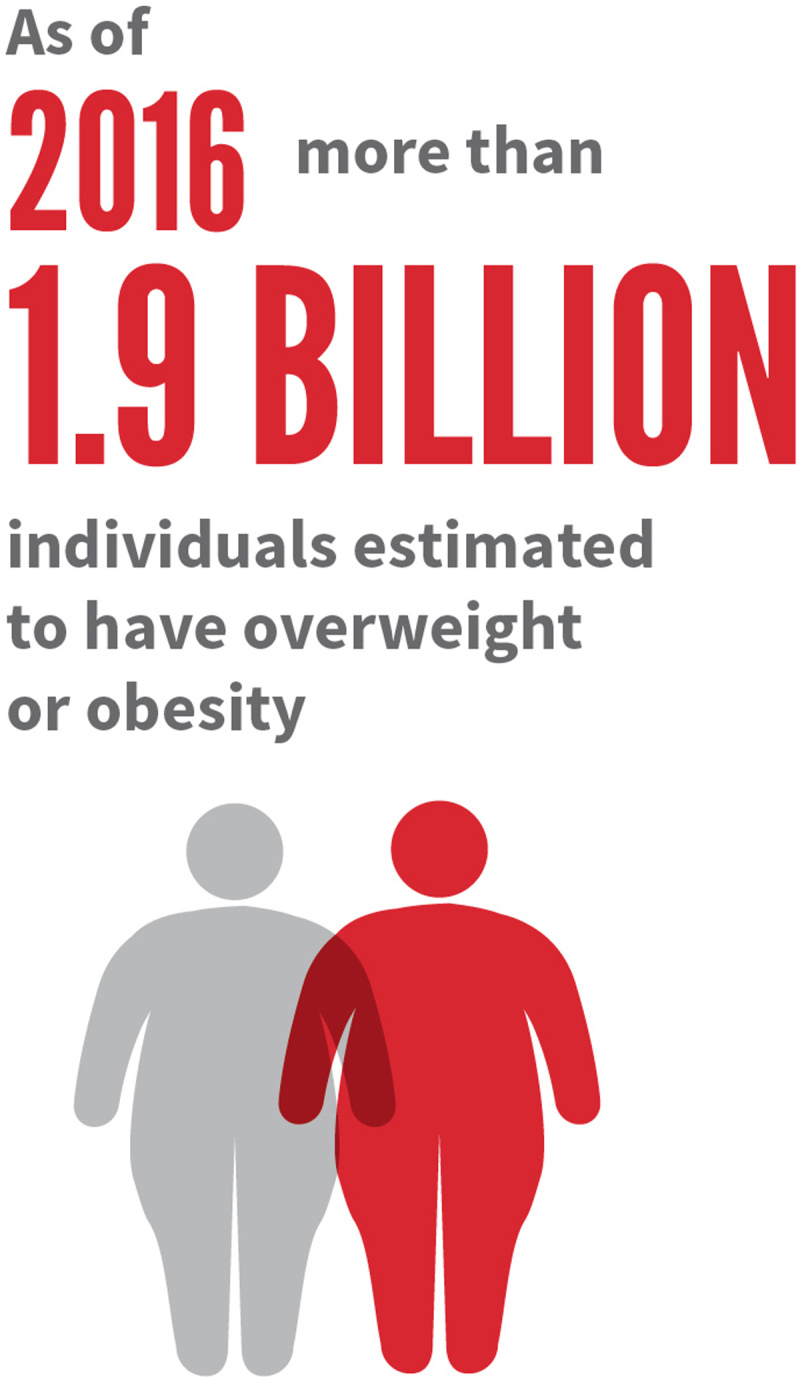


While sometimes they may have added vitamins and minerals, these generally do not justify consumption of these energy-dense, nutrient poor products.^6^ These nutrients, and the packaged foods they are found in, are all associated with diet-related noncommunicable diseases (NCDs) and are connected to the growing global obesity epidemic.^7^,^8^,^9^

Besides having a bad nutrient profile, UPP are linked to nonrecommended dietary practices, including overeating, mindless eating, and fast eating, as they increase the speed of eating rate, worsen satiety, and promote excessive energy intake.^10^,^11^

A considerable body of research highlights the large and significant impact of consuming ultra-processed foods on the major NCDs, including obesity, 12 diabetes and hypertension. The latest link was a randomized control trial conducted by Hall KD and researchers at the US National Institutes of Health (NIH) in 2019. This NIH work is supported by a wide range of over 19 cohort studies from Europe, the US, Brazil and elsewhere. These studies show that increased proportions of ultra-processed food in the diet are linked with increased risk of obesity, and heart and circulatory diseases (CVD) (diabetes, hypertension, stroke).

As of 2016, more than 1.9 billion individuals have been estimated to have overweight or obesity, and without intervention, this global health epidemic will continue to grow.

**Figure:**
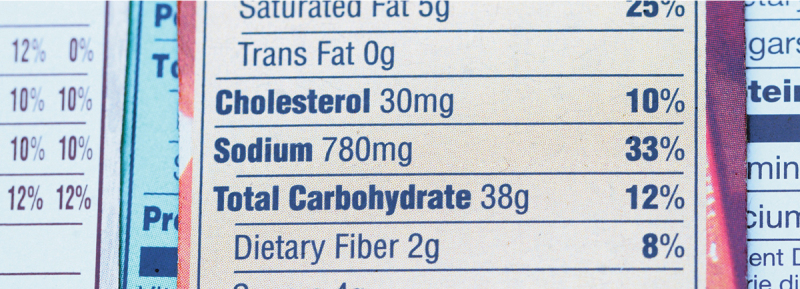


## WHAT ARE THE DIFFERENT TYPES OF FOPL?

### There is a consensus among the research community that FOPL systems can impact consumers’ choices by increasing their understanding and use of nutrition information

Several of these label systems have been shown to affect the most nutritionally at-risk populations who may have less education and nutritional knowledge.^13^,^14^,^15^,^16^,^17^ There is currently a wide variety of FOPL systems that have been implemented or are in the process of being created by governments around the world. Examples of existing systems include nutrient-specific warning labels, which alert customers when products contain excessive amounts of nutrients associated with the top risk factors for CVD and other NCDs; e.g. saturated fat and sodium; the traffic light system, which uses green, amber and red colours associated with the relative low, medium or high levels of critical nutrients in a product is another example of a nutrient-specific system; and summary indicators that rate the overall nutritional quality of a product based on algorithms that include both negative and positive nutrients and ingredients.

### Which type of label is the most effective?

While some FOPL systems are generally helpful at improving shoppers’ use and understanding of nutritional information, the success or effectiveness of a labelling system is dependent on the specific context and desired objectives.^18^ In some countries, FOPL systems seek to nudge consumers towards more healthful choices without providing nutrient-specific information. In other countries, FOPL seeks to help populations easily identify products that are excessive in critical nutrients:

- Summary score systems, such as the Nutri-Score and the Health Star Rating systems, are designed to provide consumers with an overall summary score about the (un) healthfulness of the product. It has been demonstrated that they can assist shoppers to correctly rank products from the least to the most healthful one.^19^,^20^,^21^
- Nutrition warning labels are designed to allow consumers to quickly, easily and correctly identify products that are excessive in nutrients associated with high blood pressure, high fasting blood glucose and/or overweight/obesity; i.e. sugars, totals fats, saturated fats, trans fats, and sodium. Evidence is consistent in demonstrating this system effectively fulfils this purpose, improving use and understanding of information as well as the purchase intention and purchase decisions of consumers.^22^,^23^,^24^,^25^,^26^,^27^,^28^,^29^
- Traffic light labelling systems add colours and sometimes text to the Guideline Daily Amount (GDA) scheme, providing an interpretation to the critical nutrient values as being low, medium, or high, so compared to (GDA) systems they perform better as they add interpretative elements, improving the use of information. However, conflicting valences/coding and colours can undermine the understanding of the information and weaken the impact on purchase intention or purchase decisions, so they perform worse than directive systems such as warning labels and summary scores.^30^,^31^,^32^,^33^,^34^,^35^,^36^,^37^

More research and periodic evaluations on the impacts of FOPL systems implemented at country level could help further inform the advancement of policies that can effectively contribute to improving populations’ diets.^38^

## WHAT ARE THE CHALLENGES?

### Diversity in existing systems and nutritional standards

Many countries feel that harmonising guidelines might be technically challenging due to the diversity of existing systems and nutritional profiles. Creating a global scheme would require agreeing upon a set of standards for nutritional value and measurement. Countries have expressed concern that an FOPL program might be unable to make a distinction between good or bad foods and good or bad diets due to a lack of worldwide scientific consensus on the topic.^39^

### Food industry push-back

The food manufacturing industry for the most part has been against the creation of government-endorsed FOPL systems. The Food Industry has also been more vocal in their opposition to labelling programs that are mandatory rather than voluntary. Many times, if a system is made voluntary by a country, the Food Industry will not print the labels at all or will attempt to resist and/or implement the labels very slowly. In addition to being vocal in their resistance to government-endorsed FOPL systems, food manufacturers have also tried to discourage their implementation by creating their own systems of labelling. Having the Food Industry create their own FOPL systems presents a conflict of interests, as manufacturers do not want to alert their consumers to the (un)healthfulness of their products as this transparency will impact their sales.

**Figure:**
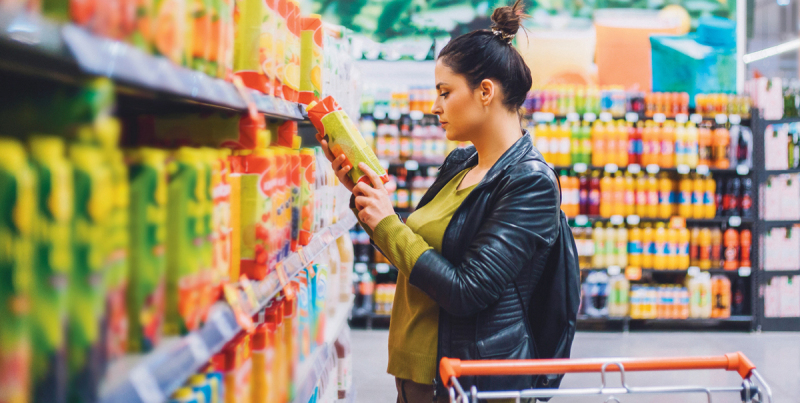


The Food Industry has been promoting a voluntary GDA FOPL system to meet the growing consumer demand. Although this means that manufacturers are labelling their products more often, research shows the GDA is the least impactful and effective FOPL system. It is hard to understand for consumers and does little to change purchasing patterns and decrease the sales of unhealthy products.

### Effectiveness for low-socioeconomic, limited education, and at-risk populations

Studies have shown that some FOPL schemes are especially useful in educating consumers on the healthfulness of pre-packaged foods, while some are very effective in helping consumers quickly, easily and correctly identify products excessive in CVD-related critical nutrients. Some schemes are good at reaching consumers with different nutrition knowledge, education and socio-economic levels,^40^,^41^,^42^ while others are considerably more useful when the consumer already possesses a baseline level of nutrition knowledge.^43^

As obesity is a more prevalent problem among lower-income groups 44 and is increasing in low- and middle-income countries,^45^ it is important to determine whether creating a FOPL policy will be able to have a serious impact on such groups.^46^

## WORLD HEALTH ORGANIZATION (WHO) GUIDING PRINCIPLES AND FRAMEWORK MANUAL FOR FRONT-OF-PACK LABELLING FOR PROMOTING HEALTHY DIETS

### The WHO guiding principles and framework manual for front-of-pack labelling for promoting healthy diets has been developed to support countries to develop, implement, and monitor and evaluate an appropriate FOPL system

The document explains that the development, implementation, and monitoring and evaluation of FOPL should be government-led, with input from key stakeholder groups. The development of FOPL should be an iterative and collaborative process. There are some key considerations and steps that need to be taken in developing an FOPL system. Various FOPL systems have already been developed by different countries. Hence, it is important to learn from the experiences of others, adapting approaches where appropriate, and taking into consideration the needs of the country while recognising the importance of global consistency. The aims, scope, and overarching and specific principles of the FOPL need to be confirmed by governments at a national and regional level, because they provide the critical framework for developing the specific details of the FOPL system. The five overarching guiding principles for FOPL that form the basis of the manual are as follows:

- Principle 1: The FOPL system should be aligned with national public health and nutrition policies and food regulations, as well as with relevant WHO guidance and Codex guidelines.
- Principle 2: A single system should be developed to improve the impact of the FOPL system.
- Principle 3: FOPL systems should not displace nutrient declarations on food packages.^47^
- Principle 4: A monitoring and review process should be developed as part of the overall FOPL system for continuing improvements or adjustments, as required.
- Principle 5: The aims, scope, and principles of the FOPL system should be transparent and easily accessible.

**Figure:**
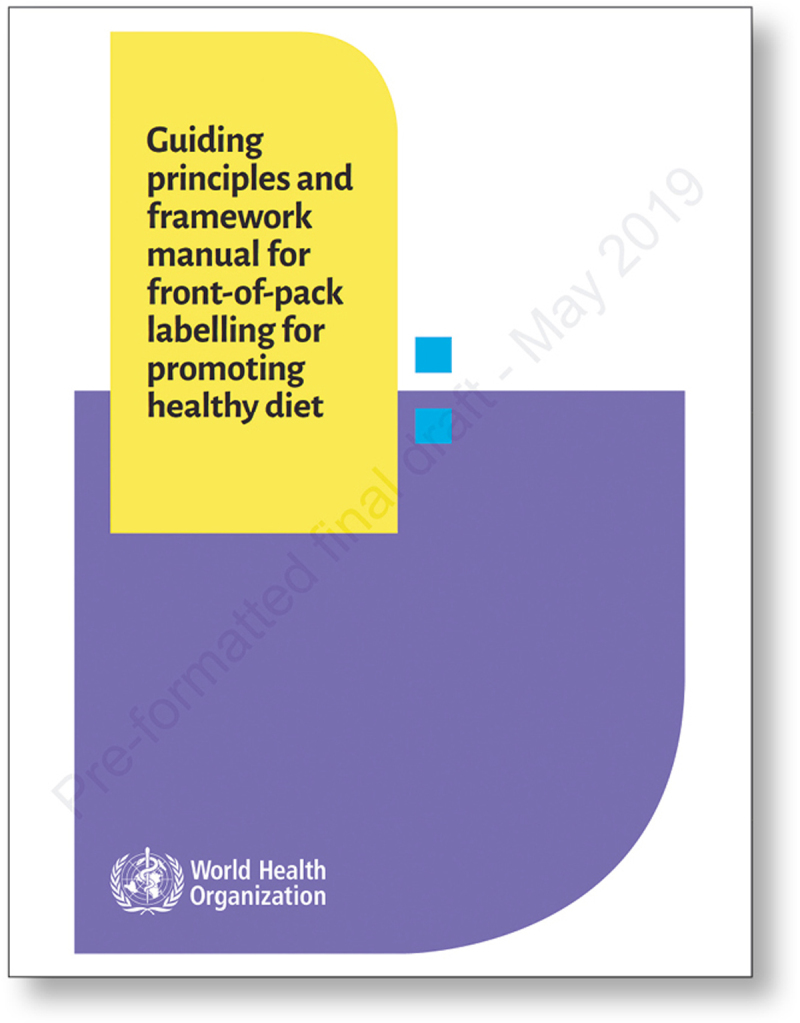


## CODEX ALIMENTARIUS COMMISSION

### The Codex Alimentarius Commission (CAC) sets international food standards that serve as a guideline for packaging and food safety for all 188 of its member nations

The CAC standards currently identify three types of nutrition labelling: nutrient declarations; nutrition and health claims; and supplementary nutrition information, which includes FOPL.^48^ Nutrient declarations are standardised listings of the nutrient content of a food or beverage, usually positioned on the back or side of the package. In 2012, Codex recommended that nutrient declarations be mandatory on food packages. However, Codex does not have clear and mandatory guidelines for FOPL.

**Figure:**
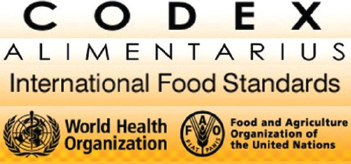


**Figure:**
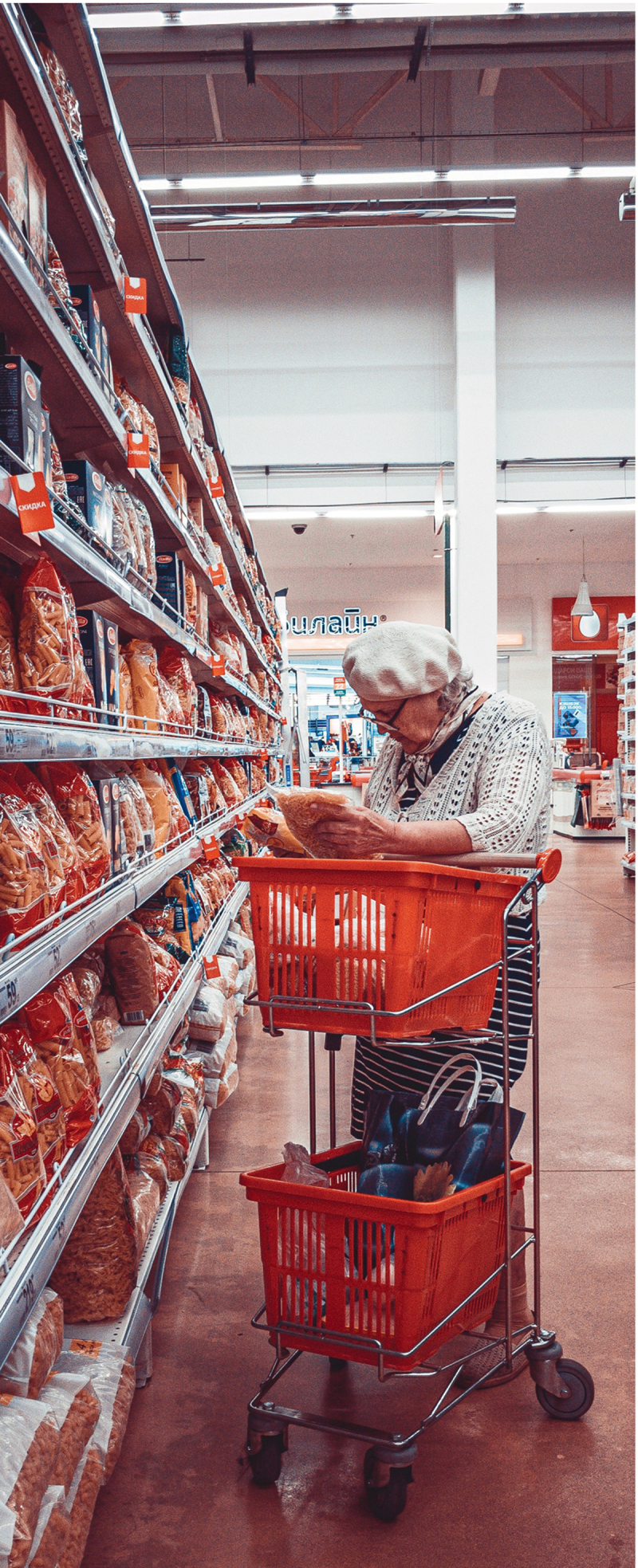


In 2016, the governments of Costa Rica and New Zealand suggested that universal regulations on FOPL should be added to the Codex standards and started the process of amending the Codex guidelines. However, at the time of publications it appears that nothing in the Guidelines will set out a specific recommended format for countries.

A draft standard of the new guidelines is currently being circulated for comments and approval, and once it has been vetted by member nations of the CAC, it will be presented by the Codex Committee on Food Labelling (CCFL) to the commission for adoption into the Codex texts.^49^

## WHAT ARE THE PROPOSED DRAFT CODEX GUIDELINES ON FOPL?

**Figure:**
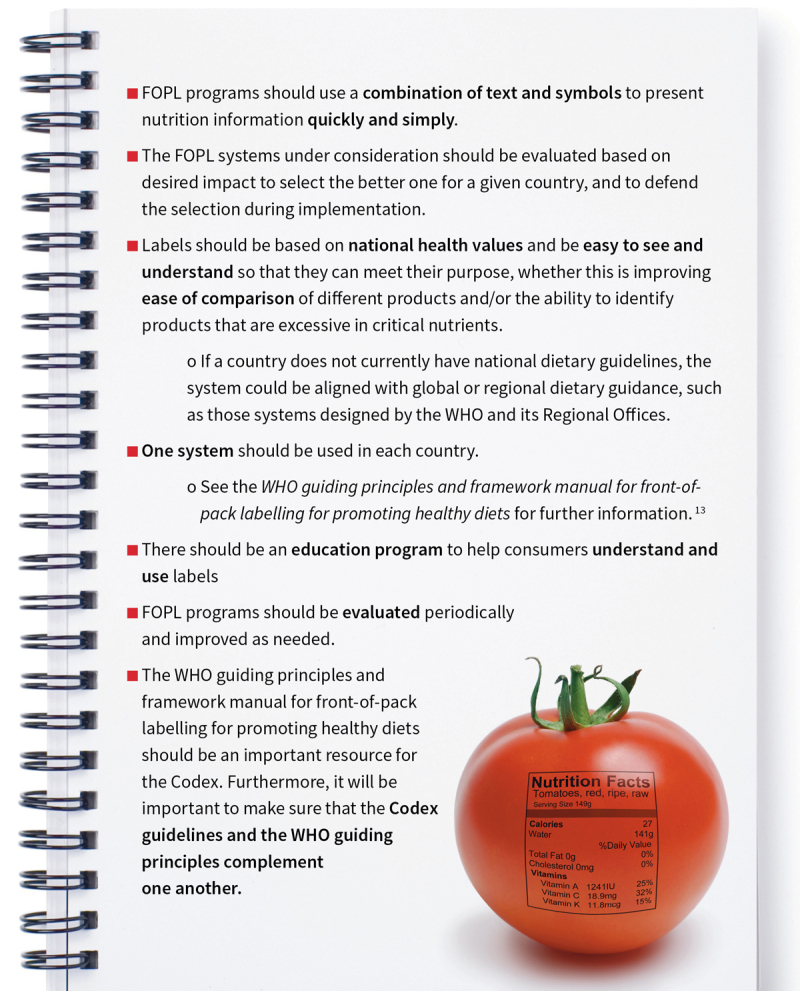


## SUCCESS STORIES

### The following FOPL policies were deemed successful based on their effectiveness in increasing consumer nutrition knowledge and/or decreasing the sale of unhealthy foods. Both policies were created by their national governments to avoid any possible conflict of interest and have had an overall positive impact on the populations that use them. These examples may be applicable to relevant regions or local contexts.

 

## CHILE

In 2012, Chile adopted some of the strongest mandatory FOPL regulations to warn consumers if products were high in either sugar, sodium, saturated fats, or calories.^50^

**Figure:**
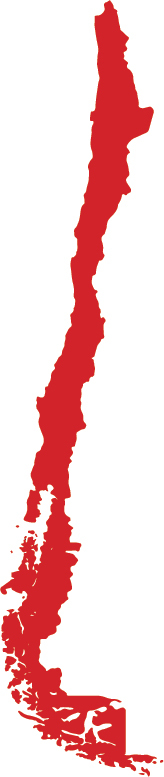


Evidence from several evaluation studies and impact assessments conducted since the legislation was enacted have demonstrated that consumers in Chile are aware of and understand the Chilean FOPL, and that they are using the labels to make decisions about food purchases.^51^,^52^ The labels are also contributing to a shift in social norms and attitudes towards purchasing nutritious food.^53^ Because the labelling system is relatively simple and has been used by all types of consumers in Chile, it has an impact across different socioeconomic and education levels, and greatly impacts the purchasing pattern of all Chileans. It has also contributed to the reformulation of some products.^54^

**Figure:**
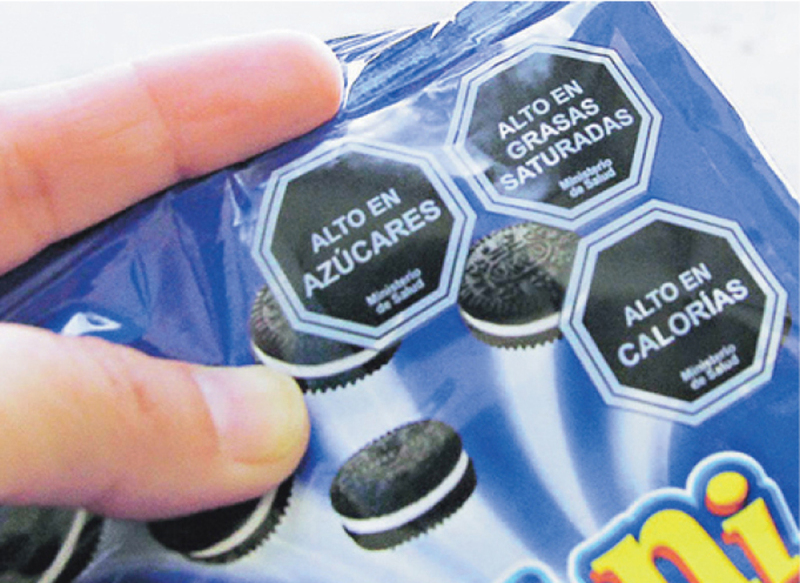
Photo credit: Chicureo Hoy SPA

## FRANCE

In France, a voluntary FOPL system was created and has been implemented since 2017. It displays a nutritional summary score of products using five colours (from green to red) and a grading system from A to E. The score is computed by accounting for the amount of positive nutrients and ingredients (fruits, vegetables and nuts, fibres, and proteins) and the product’s negative nutrient and caloric concentration (too many calories, saturated fat, sugars, and/or sodium), resulting in an overall score of the product.

**Figure:**
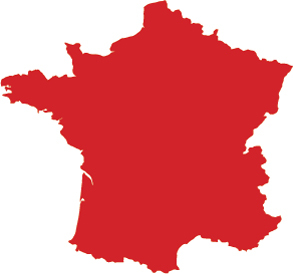


**Figure:**
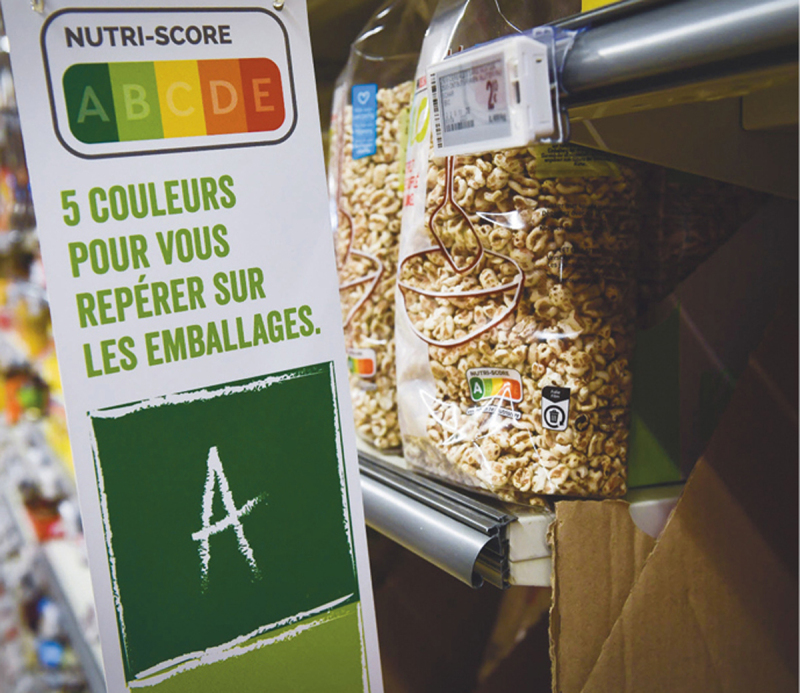
Photo credit: Laurie Dieffembacq AFP

This scheme, known as “Nutri-Score,” has been found to be particularly useful and effective for educating and informing nutritionally at-risk individuals in France. The labelling system is justified on public health grounds based on the positive impact it has had on French consumers and their overall nutrition.

## WHAT ARE THE POLICY RECOMMENDATIONS?

### While the WHO guidelines give specific recommendations for many aspects of the FOPL programme, there are some areas of the system that are flexible. This allows countries to tailor their FOPL system to the needs of their populations. Listed below are some of these flexible areas, as well as the corresponding WHF policy recommendations.

**Table:** 

Aspects to be considered	What are the WHF recommendations?	What is the reasoning?
What should be the principal message to consumers?	Consumers should avoid ultra-processed foods and instead focus on “real food”; that is, less processed, high-nutrient, preferably fresh foods.	There is a significant negative impact of consuming excessive ultra-processed foods on cardiovascular health, including obesity, diabetes, hypertension, and other conditions.
Should the system provide a nutritional summary or talk about levels of specific nutrients?	The system should be aligned with national public health and nutrition policies (dietary guidelines) and food regulations, as well as with relevant WHO guidance and Codex guidelines. It should allow for easy and quick identification of ultra-processed and processed products that have an excess of energy and key nutrients of concern, including sugar-sweetened beverages and high-fat or salty snacks, to contribute to the prevention of overweight and obesity and diet-related NCDs.	The purposes of FOPL can vary. In some countries, they seek to allow consumers to better rank products according to their healthfulness (without necessarily allowing them to identify which products are excessive in critical nutrients) and in other countries they seek to help the population easily identify products that are excessive in critical nutrients. Summary scores, such as the Nutri-Score system, meet the first purpose only, not the second. Warning labels meet the second purpose, not the first. When many purposes are pursued, it is unlikely that any one will be fully achieved. Healthy nutrients in a product can work as claims that stimulate the consumption of unhealthy products. For example, a cracker high in sodium with significant amounts of fibre should not be encouraged as a source of fibre. If this conflict is present, it will undermine the achievement of the objective. FOPL systems that are in line with or exceed the minimum requirements and recommendations of Codex and WHO are likely to be better positioned to achieve their health objectives.
Is WHF in favour of one system over than another?	WHF does not privilege one system over another and encourages governments to consider their situation and the objectives that best correspond in selecting an FOPL system. Whichever option is used, WHF believes that a comprehensive system should be put in place, rather than reliance on the less effective GDA.	WHF is aware that some countries favour summary systems (such as Nutri-Score) to help their population to be able to rank products according to their healthfulness. WHF is also aware that other countries favour warning labels because they want to help their population to easily identify products that are excessive in critical nutrients. The research on warning labels is very consistent in demonstrating that warning labels meet the regulatory objectives these countries are seeking; namely, helping consumers to easily, quickly and correctly identify products that are excessive in critical nutrients and that are linked to the three risk factors of the most burdensome diseases (high blood pressure, high fasting blood glucose, and overweight and obesity).
What foods should be labelled?	Pre-packaged processed and ultra-processed food products (UPP).	Pre-packaged processed and UPP foods apply several persuasive elements to their labels that drive consumers to purchase products that are excessive in critical nutrients and reduce the relevance of nutrition information and the capacity of consumers to make informed decisions.
Should the FOPL system be voluntary or mandatory?	FOPL systems should be mandatory.	Voluntary labelling may bias consumer perceptions towards products with labels that are potentially less healthful than products with no labels and takes longer to implement.
Who should be involved in developing the national guidelines?	National governments must be responsible for the creation of the FOPL systems in each country. Consumers, civil society/public health groups, and food manufacturers should be allowed to share their views, when appropriate, during public consultation processes.	Governments must ensure there are no conflicts of interest throughout the development of the system. Consumers, manufacturers, and public health groups should be able to provide feedback through public consultations, to ensure transparency, independence, and rigour.
What is the role of World Heart Federation and other science-based civil society organizations?	When a government is addressing its responsibilities and mandates towards reducing obesity and improving public health, civil society should support it. However, when a government does not provide the required leadership, civil society should organize and advocate for improved policies and strategies to address malnutrition in all its forms.	To advance the WHF vision of a world where heart health for everyone is a fundamental human right and a crucial element of global health justice, it is necessary to address major risk factors such as obesity and overweight. Front-of-pack labelling is an acknowledged part of an effective strategy to address diet-related risks.
What should the education program look like?	Consumer FOPL education must make individuals aware of the existence of new labels and the objectives they pursue. An effective education programme should also help consumers understand what is considered a healthy diet and how the FOPL relate to national dietary guidelines.	Consumers should understand why the labels were developed and how to read them. They should also learn what is considered a healthy diet in order to use the labels to make more nutritious purchases.
How will the system be evaluated?	The FOPL system should be regularly monitored by the national government for uptake, impact on purchasing patterns, and efficiencies and improved as necessary. Science-based civil society organizations and/or academic institutions may wish to conduct additional reviews or research that are independent of the government.	Evaluation and success can be based on improvements in consumer nutritional knowledge and changes in consumer spending on food, i.e., is there a higher rate of healthy food being purchased? Because the purposes of FOPL can vary, studies and evaluations should make sure that they evaluate results against the specific objectives of a system; studies that compare Nutri-Score systems and warning label systems cannot evaluate their respective effectiveness well if they only examine issues related to the purpose of one or the other. For example, if a study is designed to examine an FOPL system’s success by asking consumers to correctly identify which products are excessive in any of the critical nutrients, the survey would find good results for warning label systems but poor performance from Nutri-Score, simply because the latter was not designed for that purpose.

**Figure:**
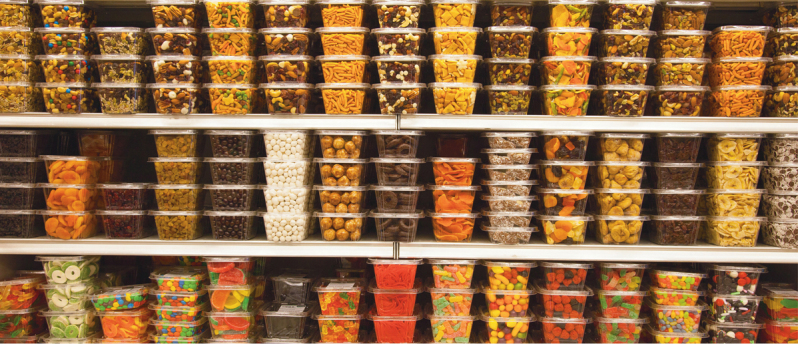


## References

[1] World Health Organization. (2017). Tackling NCDs: ‘best buys’ and other recommended interventions for the prevention and control of noncommunicable diseases World Health Organization https://apps.who.int/iris/handle/10665/259232. License: CC BY-NC-SA 3.0 IGO

[2] Poirier, P., Giles, T. D., Bray, G. A., & Hong, Y. (2005, 12 27). Obesity and Cardiovascular Disease: Pathophysiology, Evaluation, and Effect of Weight Loss. Circulation, 113(6), 898–918. Retrieved from https://doi.org/10.1161/CIRCULATIONAHA.106.171016 16380542 10.1161/CIRCULATIONAHA.106.171016

[3] American Heart Association (2018). Policy Position Statement on Food Package and Retail Shelf Icon Systems. Retrieved from: https://www.heart.org/-/media/files/about-us/policy-research/policy-positions/access-to-healthy-food/front-of-pack-labeling-policy-position-statement-2018.pdf?la=en&hash=54C1A66EFC5EC13C6FD92B48F56CF72010C7A95E

[4] Julia, C., Etilé, F., & Hercberg, S. (2018). Front-of-pack Nutri-Score labelling in France: An evidence-based policy. The Lancet Public Health, 3(4). doi: 10.1016/s2468-2667(18)30009-4 29483002

[5] Pan American Health Organization. Ultra-processed food and drink products in Latin America: Sales, sources, nutrient profiles, and policy implications Washington, D.C.: PAHO; 2019: https://iris.paho.org/bitstream/handle/10665.2/51094/9789275120323_eng.pdf?sequence=5&isAllowed=y

[6] https://www.ncbi.nlm.nih.gov/pubmed/30744710.

[7] Popkin, B. M., PhD, Willet, W., MD, DrPH., et al. THE ROLE OF AND RECOMMENDATIONS FOR FRONT-OF-PACKAGE (FOP) FOOD LABELING [Letter to Government of Uruguay].

[8] Monteiro, C.A., Cannon, G., Lawrence, M., Costa Louzada, M.L. and Pereira Machado, P. 2019 Ultra-processed foods, diet quality, and health using the NOVA classification system Rome, FAO: http://www.fao.org/3/ca5644en/ca5644en.pdf

[9] Ultra-Processing or Oral Processing? A Role for Energy Density and Eating Rate in Moderating Energy Intake from Processed Foods: Ciarán G Forde, Monica Mars, Kees de Graaf: Current Developments in Nutrition, Volume 4, Issue 3, 3 2020, nzaa019 https://academic.oup.com/cdn/article/4/3/nzaa019/5731802 32110771 10.1093/cdn/nzaa019 PMC7042610

[10] Anthony Fardet. Minimally processed foods are more satiating and less hyperglycemic than ultra-processed foods: a preliminary study with 98 ready-to-eat foods: https://pubs.rsc.org/en/content/articlelanding/2016/fo/c6fo00107f/unauth#!divAbstract 10.1039/c6fo00107f 27125637

[11] Anthony Fardet, *a Caroline Méjean, b Hélène Labouré, c Valentina A. Andreevab and Gilles Feron. The degree of processing of foods which are most widely consumed by the French elderly population is associated with satiety and glycemic potentials and nutrient profiles†: https://pubs.rsc.org/en/content/articlelanding/2017/fo/c6fo01495j/unauth#!divAbstracthttps://academic.oup.com/cdn/article/4/3/nzaa019/5731802 10.1039/c6fo01495j 28106215

[12] The Global Syndemic of Obesity, Undernutrition, and Climate Change: The Lancet Commission report. Citation Data Lancet (London, England), ISSN: 1474-547X, Vol: 393, Issue: 10173, Page: 791–846 Publication Year 2019: https://www.thelancet.com/pdfs/journals/lancet/PIIS0140-6736(18)32822-8.pdf 10.1016/S0140-6736(18)32822-8 30700377

[13] De Morais Sato P, Mais LA, Khandpur N, et al. Consumers’ opinions on warning labels on food packages: A qualitative study in Brazil. PLoS One. 2019; 14(6): e0218813 Published 2019 6 26. doi: 10.1371/journal.pone.0218813: https://www.ncbi.nlm.nih.gov/pmc/articles/PMC6594644/31242235 PMC6594644

[14] Neha Khandpur 1,*, Priscila De Morais Sato 1, Laís Amaral Mais 2, Ana Paula Bortoletto Martins 2, Carla Galvão Spinillo 3, Mariana Tarricone Garcia 2, Carlos Felipe Urquizar Rojas 3 and Patrícia Constante Jaime Are Front-of-Package Warninag Labels More Effective at Communicating Nutrition Information than Traffic-Light Labels? A Randomized Controlled Experiment in a Brazilian Sample. Nutrients 2018, 10(6), 688; 10.3390/nu10060688; Received: 4 May 2018/Revised: 21 May 2018/Accepted: 22 May 2018/Published: 28 5 2018: https://www.mdpi.com/2072-6643/10/6/688/htm PMC6024864 29843449

[15] Nieto, C., Jáuregui, A., Contreras-Manzano, A. et al. Understanding and use of food labeling systems among Whites and Latinos in the United States and among Mexicans: Results from the International Food Policy Study, 2017. Int J Behav Nutr Phys Act 16, 87 (2019). 10.1186/s12966-019-0842-1: https://ijbnpa.biomedcentral.com/articles/10.1186/s12966-019-0842-1 31623663 PMC6798377

[16] Khandpur N, de Morais Sato P, Mais LA, Bortoletto Martins AP, Spinillo CG, Garcia MT, Urquizar Rojas CF, Jaime PC. Are Front-of-Package Warning Labels More Effective at Communicating Nutrition Information than Traffic-Light Labels? A Randomized Controlled Experiment in a Brazilian Sample. Nutrients. 2018 5 28; 10(6): 688. doi: 10.3390/nu10060688 PMID: 29843449; PMCID: PMC6024864: https://www.ncbi.nlm.nih.gov/pmc/articles/PMC6024864/PMC6024864 29843449

[17] Jorge Vargas-Meza, Alejandra Jáuregui, Selene Pache o-Miranda, Alejandra Contreras-Manzano, Simón Barquera; Front-of-pack nutritional labels: Understanding by low- and middle-income Mexican consumers; Published: 11 18, 2019; 10.1371/journal.pone.0225268; https://journals.plos.org/plosone/article?id=10.1371/journal.pone.0225268 PMC6860442 31738782

[18] Front-of-package Food Labels: A Narrative Review Norman J Temple 1: https://www.ncbi.nlm.nih.gov/pubmed/31605724 10.1016/j.appet.2019.104485 31605724

[19] Ducrot, P., Méjean, C., Julia, C., Kesse-Guyot, E., Touvier, M., Fezeu, L.K., Hercberg, S. and Péneau, S., 2015 Objective understanding of front-of-package nutrition labels among nutritionally at-risk individuals. Nutrients, 7(8): https://pubmed.ncbi.nlm.nih.gov/26305255/10.3390/nu7085325 PMC4555164 26305255

[20] See: https://www.ncbi.nlm.nih.gov/pubmed/29843449 https://www.ncbi.nlm.nih.gov/pubmed/28625228 https://www.ncbi.nlm.nih.gov/pubmed/28428151

[21] Julia, C., Etilé, F., & Hercberg, S. (2018). Front-of-pack Nutri-Score labelling in France: An evidence-based policy. The Lancet Public Health, 3(4). doi: 10.1016/s2468-2667(18)30009-4 29483002

[22] Arrúa A, Machín L, Curutchet MR, Martínez J, Antúnez L, Alcaire F, et al. Warnings as a directive front-of-pack nutrition labelling scheme: comparison with the Guideline Daily Amount and traffic-light systems. Public Health Nutr 2017; 20(13): 2308–17. https://pubmed.ncbi.nlm.nih.gov/28625228/28625228 10.1017/S1368980017000866 PMC10262271

[23] Cabrera M, Machín L, Arrúa A, Antúnez L, Curutchet MR, Giménez A, Ares G. Nutrition warnings as front-of-pack labels: influence of design features on healthfulness perception and attentional capture. Public Health Nutr 2017; 2: 1–12: https://pubmed.ncbi.nlm.nih.gov/28965531/10.1017/S136898001700249X PMC10261656 28965531

[24] Sacks G, Rayner M, Swinburn B. Impact of front-of-pack ‘traffic-light’ nutrition labelling on consumer food purchases in the UK. Health Promot Int 2009; 24(4): 344–52. https://pubmed.ncbi.nlm.nih.gov/19815614/19815614 10.1093/heapro/dap032

[25] Khandpur N, de Morais Sato P, Mais LA, et al. Are Front-of-Package Warning Labels More Effective at Communicating Nutrition Information than Traffic-Light Labels? A Randomized Controlled Experiment in a Brazilian Sample. Nutrients 2018; 10(6): 688 https://pubmed.ncbi.nlm.nih.gov/29843449/10.3390/nu10060688 PMC6024864 29843449

[26] Goodman S, Vanderlee L, Acton R, Mahamad S, Hammond D. The Impact of Front-of-Package Label Design on Consumer Understanding of Nutrient Amounts. Nutrients, 2018; 10(11): 1624 https://www.ncbi.nlm.nih.gov/pmc/articles/PMC6266389/10.3390/nu10111624 PMC6266389 30400146

[27] Ministry of Health of Chile. Informe de Evaluación de la Implementación de la Ley sobre composición nutricional de los alimentos y su publicidad [Evaluation Report on the Implementation of the Law on the Nutritional Composition of Foods and its Advertising] Chile; 2017; 2018; 2019. Available at: https://www.minsal.cl/wp-content/uploads/2017/05/Informe-Implementaci%C3%B3n-Ley-20606-junio-2017-PDF.pdf; https://www.minsal.cl/wp-content/uploads/2018/05/Informe-Implementaci%C3%B3n-Ley-20606-febrero-18-1.pdf; https://www.minsal.cl/wp-content/uploads/2019/08/EVALUACION-LEY-DE-ALIMENTOS_julio-2019_02.pdf.

[28] Taillie LS, Reyes M, Colchero MA, Popkin B, Corvalán C. An evaluation of Chile’s Law of Food Labeling and Advertising on sugar-sweetened beverage purchases from 2015 to 2017: A before-and-after study. PLoS Med. 2020; 17(2): e1003015 Published 2020 2 11. doi: 10.1371/journal.pmed.1003015: https://www.ncbi.nlm.nih.gov/pmc/articles/PMC7012389/32045424 PMC7012389

[29] Taillie, L.S.; Hall, M.G.; Popkin, B.M.; Ng, S.W.; Murukutla, N. Experimental Studies of Front-of-Package Nutrient Warning Labels on Sugar-Sweetened Beverages and Ultra-Processed Foods: A Scoping Review. Nutrients 2020, 12, 569 https://www.mdpi.com/2072-6643/12/2/569/htm 10.3390/nu12020569 PMC7071470 32098363

[30] Sacks G, Rayner M, Swinburn B. Impact of front-of-pack ‘traffic-light’ nutrition labelling on consumer food purchases in the UK. Health Promot Int 2009; 24(4): 344–52. https://pubmed.ncbi.nlm.nih.gov/19815614/19815614 10.1093/heapro/dap032

[31] Ducrot P, Julia C, Méjean C, Kesse-Guyot E, Touvier M, Fezeu LK, Hercberg S, Péneau S. Impact of Different Front-of-Pack Nutrition Labels on Consumer Purchasing Intentions: A Randomized Controlled Trial. Am J Prev Med 2016; 50(5): 627–636 https://pubmed.ncbi.nlm.nih.gov/26699246/26699246 10.1016/j.amepre.2015.10.020

[32] Khandpur N, de Morais Sato P, Mais LA, et al. Are Front-of-Package Warning Labels More Effective at Communicating Nutrition Information than Traffic-Light Labels? A Randomized Controlled Experiment in a Brazilian Sample. Nutrients 2018; 10(6): 688 https://pubmed.ncbi.nlm.nih.gov/29843449/10.3390/nu10060688 PMC6024864 29843449

[33] Goodman S, Vanderlee L, Acton R, Mahamad S, Hammond D. The Impact of Front-of-Package Label Design on Consumer Understanding of Nutrient Amounts. Nutrients 2018; 10(11): 1624 https://www.ncbi.nlm.nih.gov/pmc/articles/PMC6266389/10.3390/nu10111624 PMC6266389 30400146

[34] Nyilasy G, Lei J, Nagpal A, Tan J. Color correct: the interactive effects of food label nutrition coloring schemes and food category healthiness on health perceptions. Public Health Nutr 2016; 19: 2122–2127. https://www.cambridge.org/core/journals/public-health-nutrition/article/colour-correct-the-interactive-effects-of-food-label-nutrition-colouringschemes-and-food-category-healthiness-on-health-perceptions/6A88980505E4ACF910BC01564F85F2B2# 26979588 10.1017/S1368980016000483 PMC10270970

[35] Machín L, Aschemann-Witzel J, Curutchet MR, Giménez A, Ares G. Traffic Light System Can Increase Healthfulness Perception: Implications for Policy Making. J Nutr Educ Behav 2018; 50(7): 668–674. https://pubmed.ncbi.nlm.nih.gov/29627330/29627330 10.1016/j.jneb.2018.03.005

[36] Arrúa A, Machín L, Curutchet MR, Martínez J, Antúnez L, Alcaire F, et al. Warnings as a directive front-of-pack nutrition labelling scheme: comparison with the Guideline Daily Amount and traffic-light systems. Public Health Nutr 2017; 20(13): 2308–17. https://pubmed.ncbi.nlm.nih.gov/28625228/28625228 10.1017/S1368980017000866 PMC10262271

[37] Jonathon P. Schuldt (2013) Does Green Mean Healthy? Nutrition Label Color Affects Perceptions of Healthfulness, Health Communication, 28: 8, 814–821, DOI: 10.1080/10410236.2012.725270 https://www.ncbi.nlm.nih.gov/pmc/articles/PMC7012389/23444895

[38] Taillie LS, Reyes M, Colchero MA, Popkin B, Corvalán C. An evaluation of Chile’s Law of Food Labeling and Advertising on sugar-sweetened beverage purchases from 2015 to 2017: A before-and-after study. PLoS Med. 2020; 17(2): e1003015 Published 2020 2 11. doi: 10.1371/journal.pmed.1003015 https://www.ncbi.nlm.nih.gov/pmc/articles/PMC7012389/32045424 PMC7012389

[39] Electronic Working Group Chaired by Costa Rica and Co-chaired by New Zealand (2017). DISCUSSION PAPER ON CONSIDERATION OF ISSUES REGARDING FRONT-OF-PACK NUTRITION LABELLING. Codex Committee on Food Labelling, CX/FL 17/44/7: http://www.fao.org/fao-who-codexalimentarius/sh-proxy/es/?lnk=1&url=https%253A%252F%252Fworkspace.fao.org%252Fsites%252Fcodex%252FMeetings%252FCX-714-44%252FWD%252Ffl44_07e.pdf

[40] Neha Khandpur, 1,* Priscila de Morais Sato, 1 Laís Amaral Mais, 2 Ana Paula Bortoletto Martins, 2 Carla Galvão Spinillo, 3 Mariana Tarricone Garcia, 2 Carlos Felipe Urquizar Rojas, 3 and Patrícia Constante Jaime 1 Are Front-of-Package Warning Labels More Effective at Communicating Nutrition Information than Traffic-Light Labels? A Randomized Controlled Experiment in a Brazilian Sample: https://www.ncbi.nlm.nih.gov/pmc/articles/PMC6024864/10.3390/nu10060688 PMC6024864 29843449

[41] Jorge Vargas-Meza, Alejandra Jáuregui, Selene Pacheco-Miranda, Alejandra Contreras-Manzano, Simón Barquera; Front-of-pack nutritional labels: Understanding by low- and middle-income Mexican consumers, Published: 11 18, 2019: https://journals.plos.org/plosone/article?id=10.1371/journal.pone.0225268 10.1371/journal.pone.0225268 PMC6860442 31738782

[42] Claudia Nieto, Alejandra Jáuregui, Alejandra Contreras-Manzano, Edna Arillo-Santillan, Simón Barquera, Christine M. White, David Hammond & James F. Thrasher; Understanding and use of food labeling systems among Whites and Latinos in the United States and among Mexicans: Results from the International Food Policy Study, 2017; International Journal of Behavioral Nutrition and Physical Activity volume 16, Article number: 87 (2019) Cite this article: https://ijbnpa.biomedcentral.com/articles/10.1186/s12966-019-0842-1 31623663 10.1186/s12966-019-0842-1 PMC6798377

[43] Ducrot, P., Méjean, C., Julia, C., Kesse-Guyot, E., Touvier, M., Fezeu, L.K., Hercberg, S. and Péneau, S., 2015 Objective understanding of front-of-package nutrition labels among nutritionally at-risk individuals. Nutrients, 7(8): https://pubmed.ncbi.nlm.nih.gov/26305255/10.3390/nu7085325 PMC4555164 26305255

[44] Ducrot, P., Méjean, C., Julia, C., Kesse-Guyot, E., Touvier, M., Fezeu, L.K., Hercberg, S. and Péneau, S., 2015 Objective understanding of front-of-package nutrition labels among nutritionally at-risk individuals. Nutrients, 7(8) 10.3390/nu7085325 PMC4555164 26305255

[45] Tara Templin, Tiago Cravo Oliveira Hashiguchi, Blake Thomson, Joseph Dieleman, Eran Bendavid; The overweight and obesity transition from the wealthy to the poor in low- and middle-income countries: A survey of household data from 103 countries Published: 11 27, 2019; 10.1371/journal.pmed.1002968: https://journals.plos.org/plosmedicine/article?id=10.1371/journal.pmed.1002968 PMC6880978 31774821

[46] Poirier, P., Giles, T. D., Bray, G. A., & Hong, Y. (2005, 12 27). Obesity and Cardiovascular Disease: Pathophysiology, Evaluation, and Effect of Weight Loss. Circulation, 113(6), 898–918. Retrieved from https://doi.org/10.1161/CIRCULATIONAHA.106.171016 16380542 10.1161/CIRCULATIONAHA.106.171016

[47] This statement is a clarification of the WHO 3rd principle, which says “Mandatory nutrient declarations on food packages are a prerequisite for FOPL systems.” It has been modified here because many countries do not yet have nutrient declarations that are mandatory (back-of-pack panels). These countries should be able to advance FOPL in any event. By saying that the nutrient declaration is a “prerequisite”, it is suggesting that one must happen before the other, which is not necessary, as the country may, for example, advance both at the same time as part of a single regulatory process.

[48] Codex Alimentarius Commission. Guidelines on nutrition labelling CAC/GL 2-1985 Geneva: Food and AgricultureOrganization of the United Nations (FAO) and World Health Organization (WHO); 2015.

[49] Electronic Working Group Chaired by Costa Rica and Co-chaired by New Zealand, (2019). PROPOSED DRAFT GUIDELINES ON FRONT-OF-PACK NUTRITION LABELLING. Codex Committee on Food Labelling, http://www.fao.org/fao-who-codexalimentarius/sh-proxy/en/?lnk=1&url=https%253A%252F%252Fworkspace.fao.org%252Fsites%252Fcodex%252FMeetings%252FCX-714-45%252Fdocuments%252Ffl45_06e_final.pdf

[50] Araya, Sebastián, Elberg, Andrés, Noton, Carlos and Schwartz, Daniel, (2018), Identifying Food Labeling Effects on Consumer Behavior, No 338, Documentos de Trabajo, Centro de Economía Aplicada, Universidad de Chile, https://EconPapers.repec.org/RePEc:edj:ceauch:338.

[51] Ministry of Health of Chile. Informes de Evaluación de la Implementación de la Ley sobre composición nutricional de los alimentos y su publicidad [Evaluation Reports on the Implementation of the Law on the Nutritional Composition of Foods and its Advertising] Chile; 2017; 2018; 2019. Available at: https://www.minsal.cl/wp-content/uploads/2017/05/Informe-Implementaci%C3%B3n-Ley-20606-junio-2017-PDF.pdf; https://www.minsal.cl/wp-content/uploads/2018/05/Informe-Implementaci%C3%B3n-Ley-20606-febrero-18-1.pdf; https://www.minsal.cl/wp-content/uploads/2019/08/EVALUACION-LEY-DE-ALIMENTOS_julio-2019_02.pdf.

[52] Taillie LS, Reyes M, Colchero MA, Popkin B, Corvalán C. An evaluation of Chile’s Law of Food Labeling and Advertising on sugar-sweetened beverage purchases from 2015 to 2017: A before-and-after study. PLoS Med. 2020; 17(2): e1003015 Published 2020 2 11. doi: 10.1371/journal.pmed.1003015: https://www.ncbi.nlm.nih.gov/pmc/articles/PMC7012389/32045424 PMC7012389

[53] Correa, T., Fierro, C., Reyes, M. et al. “Responses to the Chilean law of food labeling and advertising: exploring knowledge, perceptions and behaviors of mothers of young children”. Int J Behav Nutr Phys Act 16, 21 (2019). 10.1186/s12966-019-0781-x 30760273 PMC6375144

[54] Ministry of Health of Chile. Informes de Evaluación de la Implementación de la Ley sobre composición nutricional de los alimentos y su publicidad [Evaluation Reports on the Implementation of the Law on the Nutritional Composition of Foods and its Advertising] Chile; 2017; 2018; 2019. Available at: https://www.minsal.cl/wp-content/uploads/2017/05/Informe-Implementaci%C3%B3n-Ley-20606-junio-2017-PDF.pdf; https://www.minsal.cl/wp-content/uploads/2018/05/Informe-Implementaci%C3%B3n-Ley-20606-febrero-18-1.pdf; https://www.minsal.cl/wp-content/uploads/2019/08/EVALUACION-LEY-DE-ALIMENTOS_julio-2019_02.pdf

